# Lemierre’s Syndrome in a Pediatric Patient: A Case Report

**DOI:** 10.7759/cureus.48712

**Published:** 2023-11-13

**Authors:** Maitham J Aljubran, Sarah M Alhammad, Zainab A Alghanem, Hussain J Aljubran, Zainab S Aljizeeri, Walaa Aldairam

**Affiliations:** 1 Pediatric Department, Maternity and Children Hospital, Al-Ahsa, SAU; 2 College of Medicine, Imam Abdulrahman Bin Faisal University, Dammam, SAU

**Keywords:** necrobacillosis, post-anginal sepsis, jugular vein suppurative thrombophlebitis, fusobacterium necrophorum, lemierre's syndrome

## Abstract

Lemierre’s syndrome (LS) is a rare and potentially life-threatening complication of an oropharyngeal infection, resulting in the presence of septic thrombophlebitis in the internal jugular vein. This condition is most commonly caused by *Fusobacterium necrophorum*, with a prevalence of one case per million people annually. LS is more commonly seen in adolescents or young adults, but it can also occur in children. Despite its rare occurrence, prompt diagnosis of this condition and the initiation of treatment are crucial to preventing fatal complications. In this study, we report a unique case of a previously healthy seven-year-old male with LS who presented with fever, left-sided neck pain, and swelling.

## Introduction

Lemierre’s syndrome (LS), also referred to as jugular vein suppurative thrombophlebitis, postanginal sepsis, and necrobacillosis, was initially reported by Courmont and Cade in 1900. It was later best characterized by Andre Lemierre when he reported on 20 patients with LS in 1936 [1,2]. LS is a rare medical condition that affects one in one million people annually, with 90% of the reported cases involving patients aged 19-22 years [3,4]. However, LS has also been reported in pediatric patients (< 18 years old) [5-8]. This condition is distinguished by the presence of septic thrombophlebitis in the internal jugular vein (IJV). LS typically begins with an oropharyngeal infection and frequently involves inflammation within the vein’s wall, infected thrombus within the lumen, surrounding soft tissue inflammation, persistent bacteremia, and septic emboli [9]. *Fusobacterium necrophorum* is the most common anaerobic bacterium linked to LS. These bacteria belong to the *Bacteroidaceae* family and are non-motile, non-spore-forming, and obligate anaerobic Gram-negative rods [10]. Although LS has shown a steady decline in recent years with the use of penicillin to treat oropharyngeal infections once indicated, it is crucial to diagnose and treat this condition quickly, as it has a mortality rate ranging from 5% to 18% [11,12]. The purpose of this case report is to describe a unique case of a pediatric patient with LS to highlight the significance of timely LS diagnosis and treatment.

## Case presentation

A seven-year-old male without a significant past medical history presented to the pediatric emergency department (PED) with a five-day history of fever, left-sided neck pain, and swelling. Two days preceding admission, he was noticed to have dysphagia and torticollis. There was no history of a recent infection, and the boy was not on any regular medication. His family history was unremarkable.

Upon arrival at the emergency department, the patient was conscious, alert, and looking well. He had an average build (weight 23 kg, height 121 cm - both below the 75th percentile). However, he was found to be febrile (38.7 °C), tachycardic (123 beats/min), normotensive (95/60 mmHg), breathing 24 breaths/min, and saturating 96% in room air.

On physical examination, the patient had left-sided neck swelling with decreased cervical passive and active range of motion secondary to pain. The left-sided neck swelling was firm, smooth, regular, tender, not mobile, and 3*5 cm in size. In addition, he had multiple tender and enlarged bilateral cervical lymphadenopathy with no overlying skin changes. His ear, nose, and throat (ENT) examination was insignificant, and the rest of his systemic examination was unremarkable. 

Table 1 shows the initial laboratory tests drawn in the PED, which were significant for an elevated inflammatory marker (ESR: 97 mm/first hr), normocytic anemia (HB: 9.1 g/dL), elevated liver enzymes (AST: 59, ALT: 61 U/L), and hypoalbuminemia (24.2 g/L).

**Table 1:** Initial laboratory findings at the time of diagnosis.

Tests	Reference range	Patient values
Complete blood count		
White blood cell (WBC)	5-15.5 x 10^3^	9.9
Hemoglobin	11.5-12.5 g/dL	9.1
Platelet	150-350 x10^3 ^/mL	296
Erythrocyte sedimentation rate (ESR)	0-10 mm/1^st^ hr	97
Coagulation profile		
Prothrombin time (PT)	11.7-15.1 sec	13
Partial thromboplastin time (PTT)	31.8-43.7 sec	34.1
International normalized ratio (INR)	0.87-1.20	1.09
Serum biochemistry		
Blood urea nitrogen (BUN)	3.2-7.9 mmol/L	2.8
Serum creatinine	27.4-53.93 mmol/L	46.37
Sodium (Na)	135-145 mmol/L	137
Potassium (K)	3.4-4.7 mmol/L	3.7
Chloride (Cl)	101-107 mmol/L	97
Aspartate transaminase (AST)	21-44 U/L	59
Alanine transaminase (ALT)	9-25 U/L	61
Albumin	38-47 g/L	24.2
Calcium (Ca)	2.3-2.6 mmol/L	2.18
Magnesium (Mg)	0.86-1.17 mmol/L	0.83
Phosphate (PO4)	1.3-1.9 mmol/L	1.4
Blood culture	-	No growth after 5 days incubation

**Figure 1:**
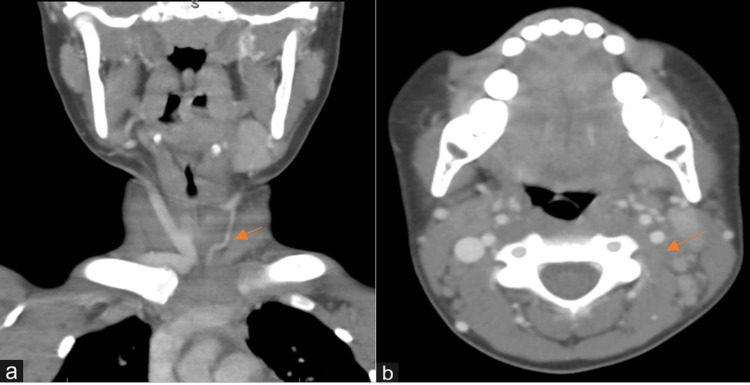
A neck computed tomography slices showing areas of thrombophlebitis (arrows) of left internal jugular vein. (a) Coronal view of the neck CT. (b) Horizontal section of the neck CT.

Neck ultrasonography showed multiple enlarged cervical lymph nodes on both sides of the neck with the largest measured as 11 mm, with no evidence of necrosis or calcification. There was evident thrombosis of the left IJV associated with perivascular fat stranding/inflammatory process consistent with left IJV thrombophlebitis. A neck computerized tomography (CT) scan with contrast confirmed the findings, which showed non-opacified proximal left IJV with no focal mass lesion or abscess collection (Figures 1a, 1b). The patient was classified as a high-risk patient to develop thrombosis, so he was admitted to the Pediatric Intensive Care Unit (PICU) for close monitoring and management.

During his PICU admission, the patient had a workup for a hypercoagulable state and autoantibodies to rule out systemic lupus erythematosus (SLE). All results were negative, except for a high D-dimer (1.08) (Table 2). The patient was kept on low molecular weight heparin (enoxaparin) and IV ceftriaxone and clindamycin. After one day of observation, the patient remains stable and transferred to the pediatric ward.

**Table 2:** Laboratory results at the time of admission to the PICU.

Tests	Reference range	Patient results
Protein C	0.64-1.25 U/mL	0.69
Protein S	0.64-1.54 U/mL	0.96
Anti-thrombin III	0.90-1.31 U/mL	1.16
Factor V Leiden	120-300 sec	172.8
Fibrinogen	1.99-1.09 g/L	431
D-Dimer	0.10-0.56 μ/mL	1.08
HB electrophoresis		
HBA2	2%-3%	2.4
HBA	95%-98%	97.6

The course of his hospital admission to the ward showed no complications and no signs of metastatic infections. The blood culture drawn in the PED on day 1 of admission showed no growth. Echocardiography was normal.

Magnetic resonance angiography (MRA) of the neck and chest was planned; however, the patient was discharged home after four days and before the MRA could be performed. He was discharged against medical advice (DAMA) after improvement of clinical manifestations, and he planned to complete a six-week course of oral antibiotics and enoxaparin. Three days later, the patient visited the outpatient clinic and was doing fine, with no complaints. He planned to continue to follow up with the infectious disease clinic and the hematology clinic.

## Discussion

LS is a rare medical condition that occurs when an infection in the oropharyngeal space leads to septic thrombophlebitis in the IJV [13]. Although LS is more commonly seen in adolescents or young adults, it could also be present in the pediatric age group, as was seen in our case [5-8]. Diagnosing LS is challenging because it is rare and lacks distinct symptoms. In addition, this rare condition is related to severe complications that can result from oropharyngeal infections, as the disease may progress rapidly and lead to sepsis, pneumonia, or other life-threatening complications in some cases [9].

The absence of agreement regarding the precise numerical description of a genus poses difficulties when it comes to accurately determining the organism accountable for LS within the population. Although there is no evidence to suggest an underlying organism that increases the occurrence of LS, there has been a rise in the number of reported cases which can be attributed to various factors, including an excessive dependence on negative rapid strep tests, a lack of awareness, and changes in the prescription of antibiotics. Furthermore, the resurgence of LS may be linked to the decrease in the use of penicillin, which increases the potential for the disease to develop. Previous studies show that *F. necrophorum* is the responsible organism of LS in over 70% of cases [10]. Nevertheless, in rare cases, other bacteria, such as *Streptococcus*,* Staphylococcus*, or *Bacteroides*, may be responsible [9]. *F. necrophorum* takes two to seven days to show growth in blood cultures; however, our patient’s blood culture showed no growth.

The diagnosis of LS in children can be challenging, as the condition is rare and may not initially be suspected, similar to our case. During the initial presentation, the clinical manifestations could vary depending on the site of the infection. However, in more than 80% of patients, there is a presence of fever, and about 50% of patients experience abdominal pain, nausea, and vomiting [14]. Similarly, our case presented with fever, left-sided neck pain, and swelling as the primary manifestations of his condition.

The confirmation of LS is typically accomplished by conducting laboratory tests and imaging instead of relying on the patient’s clinical symptoms. After initial laboratory investigations, a CT scan with contrast is considered the primary imaging in a patient above 14 years of age. For children under 14 years of age, an ultrasound of the neck may be ordered before a CT scan with contrast due to concerns about the risk of radiation associated with CT scan in this age group. Diagnosis is confirmed with imaging that shows jugular vein thrombosis with appropriate clinical symptoms. This imaging findings triad usually includes three signs: IJV thrombosis, pulmonary septic emboli, and ipsilateral pharyngeal fullness [15]. In this case, a CT scan with contrast was performed, and it revealed thrombosis of the left IJV associated with the perivascular fat stranding/inflammatory process. While MRI is also an excellent method for visualizing all anatomic structures, as well as thrombosis and/or septic emboli, it is expensive and usually not readily available [5,9].

The treatment of LS in children typically involves aggressive intravenous antibiotic therapy. According to some studies, a combination of high-dose penicillin and metronidazole or monotherapy with clindamycin is recommended as a regimen [16]. However, our patient was treated with an IV ceftriaxone and clindamycin regimen. The appropriate course for antibiotic treatment has been a topic of debate. According to the literature, the duration of treatment can fall anywhere between nine and 128 days, with the typical range being three to six weeks; thus, our patient was treated with a six-week course [14]. Additionally, supportive care, such as oxygen therapy or mechanical ventilation, may be necessary in severe cases, and surgical intervention may be necessary to drain abscesses or remove infected tissue. Although an anticoagulant was prescribed in our case, the role of this drug in LS treatment is still controversial, and no randomized clinical trial supports its use [9,13].

After a child has recovered from LS, it is important that they receive appropriate follow-up care to monitor their health and prevent potential long-term complications, such as neurologic deficits and debilitating conditions. Overall, the follow-up care recommended for children with LS depends on their individual health status and the severity of their infection. It is important for parents and caregivers to work closely with their child’s healthcare provider to develop a personalized follow-up plan, which includes regular visits to their pediatrician and an infectious disease specialist, ENT specialist, and hematology specialist, as needed. It is critical to repeat imaging (to monitor the child’s recovery and detect any potential residual abscesses or thrombosis), provide education to the parents or caregivers regarding the child’s condition, and offer mental health support [17].

## Conclusions

LS is a potentially life-threatening complication of an oropharyngeal infection that could ultimately result in serious systemic damage. Although LS is a rare condition that is seen more commonly in adolescents or young adults, our study highlighted that this condition should also be suspected in the pediatric age group. Early recognition and treatment are needed to reduce the subsequent mortality related to the condition. Our patient received the recommended medical management, including antibiotics and an anticoagulant, and was discharged in stable condition after four days in the hospital when his family requested a DAMA.
